# Time-Varying Functional Connectivity of Rat Brain during Bipedal Walking on Unexpected Terrain

**DOI:** 10.34133/cbsystems.0017

**Published:** 2023-03-29

**Authors:** Honghao Liu, Bo Li, Pengcheng Xi, Yafei Liu, Fenggang Li, Yiran Lang, Rongyu Tang, Nan Ma, Jiping He

**Affiliations:** ^1^ School of Mechatronical Engineering, Beijing Institute of Technology, Beijing 100081, China.; ^2^ School of Information and Communication Engineering, North University of China, Taiyuan 038507, China.; ^3^ Beijing Advanced Innovation Center for Intelligent Robots and Systems, Beijing Institute of Technology, Beijing 100081, China.; ^4^ Department of Engineering, Lancaster University, Lancaster LA1 4YW, UK.

## Abstract

The cerebral cortex plays an important role in human and other animal adaptation to unpredictable terrain changes, but little was known about the functional network among the cortical areas during this process. To address the question, we trained 6 rats with blocked vision to walk bipedally on a treadmill with a random uneven area. Whole-brain electroencephalography signals were recorded by 32-channel implanted electrodes. Afterward, we scan the signals from all rats using time windows and quantify the functional connectivity within each window using the phase-lag index. Finally, machine learning algorithms were used to verify the possibility of dynamic network analysis in detecting the locomotion state of rats. We found that the functional connectivity level was higher in the preparation phase compared to the walking phase. In addition, the cortex pays more attention to the control of hind limbs with higher requirements for muscle activity. The level of functional connectivity was lower where the terrain ahead can be predicted. Functional connectivity bursts after the rat accidentally made contact with uneven terrain, while in subsequent movement, it was significantly lower than normal walking. In addition, the classification results show that using the phase-lag index of multiple gait phases as a feature can effectively detect the locomotion states of rat during walking. These results highlight the role of the cortex in the adaptation of animals to unexpected terrain and may help advance motor control studies and the design of neuroprostheses.

## Introduction

In the natural environment, animals often encounter various complex terrains when walking. Affected by environmental factors, such as a low-light environment, animals may not be able to visually detect changes in terrain when walking, making animals need to adjust their movements to adapt to unexpected terrain changes. Compared with normal walking, this unpredictable movement may cause considerable differences in the motor control mechanism. A previous study showed that humans take more cautious strategies when walking on unpredictable uneven terrain, such as increasing step length and reducing step width [1]. In addition, when subjects accidentally touched an obstacle during walking, they would choose different coping strategies according to the movement state of their limbs. For example, when a leg touches an obstacle in the process of swinging, the swinging leg will be immediately lifted up and over the obstacle in the early stage and be placed in front of the obstacle in the later stage [2]. It is worth mentioning that adapting to unexpected terrain seems to be not only an instinctive reaction, and its process was also influenced by previous experience [3,4]. With the development of neuroscience, more and more studies have shown that the brain plays an important role in animals' responses to unexpected disturbances. A recent study showed that the spectral power of the premotor cortex and supplementary motor area increased significantly when humans encountered unexpected obstacles while walking or running on a treadmill [5]. When subjects unexpectedly lost their balance during walking, the theta-band energy in multiple brain regions, such as the anterior cingulate and parietal, increased significantly [6]. When the sudden acceleration of the support plane disturbs the posture stability of the subjects, the activities of some cerebral cortex (such as the anterior prefrontal cortex) change differently according to the coping strategies taken by the subjects [7]. Some animal experiments have shown that when the standing platform suddenly tilts, the motor cortex become more active [8,9]. However, these studies mainly focused on analyzing the activation of each brain region and did not analyze the interaction between brain regions. The study of the interaction between cortices can help us better understand the role of the brain in the process of animal adaptation to unexpected terrain.

Brain connectivity estimation aims to describe the interaction between the cortex, including structural connectivity, functional connectivity, and effective connectivity [10]. Structural connectivity refers to the anatomical connection between brain regions [11]; functional connectivity was defined as the temporal correlation between spatially remote neurophysiologic events [12]; and effective connectivity describes the direct or indirect influence of one nervous system on another, which can be derived from functional connectivity [13]. Functional connectivity was considered the most central and challenging of the 3 concepts of brain connectivity and has been widely used in neuroscience research [14]. Currently, most network-based studies use the data of an entire scan session to build a static brain function network, which can obtain the brain connectivity characteristics of the whole process. However, walking was a cycle process consisting of stance and swing phases, and the activity of the cerebral cortex couples with gait phases [15,16]. Therefore, the static function network may not be able to capture changes in brain connectivity with gait phases. Some studies analyze the time-varying properties of networks during cognitive tasks and motor imagery by scanning the data using sliding time windows and estimating the functional connectivity within the window [17,18]. However, these studies did not apply this dynamic analysis method to the actual walking process. Considering the continuity of the walking process, analyzing the network properties on a time scale may be more helpful in understanding the cortical information interaction process during walking.

As mentioned above, our current understanding of the information interaction among brain regions in animal adaptation to unexpected terrain was limited. This study aimed to investigate animals' dynamic brain network properties when encountering unexpected terrain while walking. To address this question, we used rats as the model and built a posture-restrained platform based on a treadmill to help the rat walk in a bipedal posture. The rats' vision was blocked to remove all visual cues on the upcoming change in the terrain, and an uneven area was randomly placed on the treadmill belt to simulate the unexpected complex terrain. An implantable multimodal recording platform equipped with high-speed cameras was used to simultaneously record electroencephalography (EEG) signals and behavioral data in rats. Whole-brain EEG signals from 6 rats were scanned by time windows, and functional connectivity within the window was quantified by phase-lag index (PLI). Furthermore, the global and local metrics of the functional network were calculated on the basis of PLI. Finally, support vector machine (SVM), naive Bayes (NB) classifier, and K-nearest neighbor (KNN) classifier were used to classify rat motion states based on the PLI matrix, global metrics, and local metrics. We hypothesize that dynamic functional connectivity of the rat brain varied with gait phase and terrain conditions. Based on the differences in the time-varying function connectivity, the locomotion state of the rat can be detected.

## Materials and Methods

### Animals

**Fig. 1.**
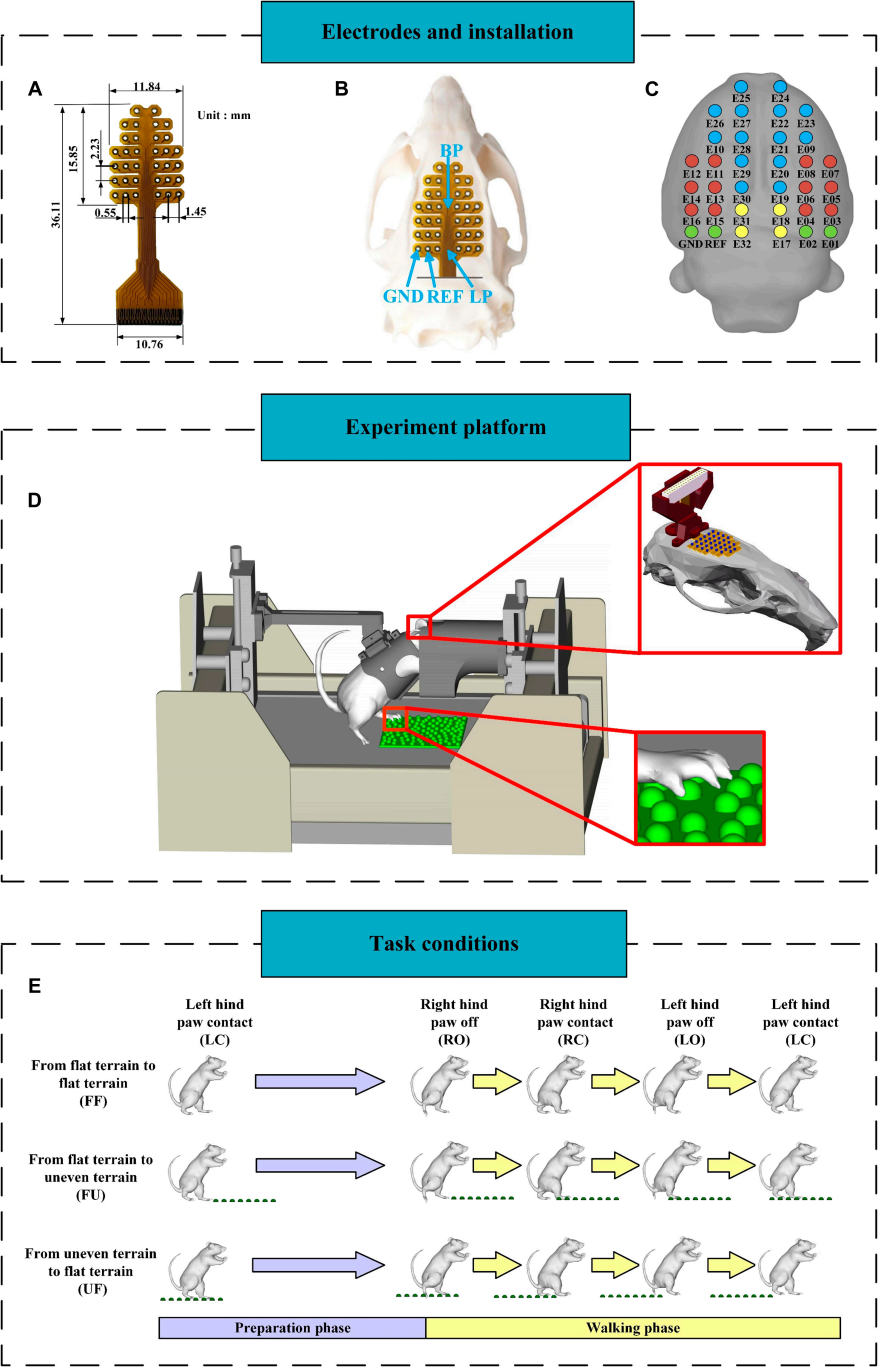
Experimental setup and task conditions. (A) The 32-channel flexible electrode arrays were used in this study. (B) Location of the electrode array on the surface of the rat skull. BP represents the bregma point; LP represents the lambda point; GND represents the ground electrode; REF represents the reference electrode. (C) The putative electrode positions on the rat’s brain surface, as determined by Brainstorm3. The colors of the electrodes correspond to the different brain regions (blue, somatomotor area; red, somatosensory area; yellow, retrosplenial area; green, visual area). (D) Platform used in this study. Rats walked on a treadmill in a bipedal posture with the help of a suspension device, and a black tube was used to block all frontal visual cues on the upcoming terrain. A self-made shell was installed on the rat skull to protect and fix the interface of the electrode array. A removable uneven area was randomly placed on the treadmill belt. (E) Task conditions in this study.

The experiments were conducted on 6 male Sprague Dawley rats (aged 8 to 10 weeks and weighing 200 to 250 g). Each rat was individually maintained in a ventilated animal facility with 12-h light/12-h dark cycles. The temperature (25 ± 2 °C), humidity, and ventilation conditions in the cage were appropriate, and food and water were provided ad lib. Rats were trained to walk in bipedal posture after 1 week of dietary control, and food rewards will be given on the basis of the performance of the rats during training. Each rat was trained for about half an hour per day. After 2 weeks of training, a home-designed 32-channel flexible electrode array (Fig. 1A) was surgically fixed on the surface of the rat skull (see [19] for surgical details). After 1 week of recovery, the rats continued to train for 2 weeks and began the formal experiment. All animal procedures were approved by the Institutional Animal Care and Use Committee of the Beijing Institute of Technology.

### Experimental procedures

Figure 1D shows the platform used in this study. Rats walked on a moving treadmill belt (6.5 to 7.5 cm/s) in a bipedal posture with the help of a suspension device, and a black tube was used to block all frontal visual cues on the upcoming terrain. An 80-fps camera was placed on each side of the treadmill to record images of the rats. First, the rat stands still on the treadmill for 10 s, then the treadmill starts, and the rat walks on a flat terrain for 20 s. Next, the rat walks for another 30 s, during which we randomly choose to place or not to place an uneven area (the size of the area was 11 × 11 cm, and hard hemispherical protrusions with a diameter of 10 mm were randomly distributed in it) on the treadmill belt in front of the rat. After that, it was regarded as the end of a trial, and the uneven area was removed. In this process, the placement time of uneven area was also random, and rats were able to pass through the uneven area and completely transition to the flat terrain. It was worth noting that uneven areas did not support continuous walking of rats (rats can leave the uneven terrain in the next step after a complete transition to the uneven terrain). There was a 30-s rest time between each trail, and each rat can complete 50 to 60 trails (about 25 to 30 trials with uneven area) on average every day.

Three types of locomotion tasks (Fig. 1E) were performed by the rats in the experiment: (a) the rat walked from the flat terrain to the flat terrain (FF); (b) the rat walked from the flat terrain to the uneven terrain (FU); and (c) the rat walked from the uneven terrain to the flat terrain (UF). In addition, 4 events were defined on the basis of the rat's gait: right hind paw coming off the ground (RO), right hind paw contacting the ground (RC), left hind paw coming off the ground (LO), and left hind paw contacting the ground (LC). Benefiting from the slow speed of the treadmill belt, the rats can stand on both hind paws for a period after completing a continuous stride (e.g., RO→RC→LO→LC) to prepare for the next continuous stride. Therefore, we defined the period before the rat stride continuously as the preparation phase and the process of striding continuously as the walking phase. In this study, the right hind paw of the rat was selected as the leading paw, so the preparation phase was from event LC to RO, the walking phase was from event RO to event LC, and the complete gait cycle was defined as LC→RO→RC→LO→LC. We used Simi Motion v9.2.2 (Simi Inc., Unterschlessheim, Germany) kinematics analysis software to extract gait events from rat walking videos to segment different gait phases and manually mark them in EEG signals. By averaging the time interval of each gait phase for all rats under all task conditions (1,846 complete gait cycles in total, each rat contributed a similar number of gait cycles under each condition), the duration of the preparation stage was 747 ± 38 ms (mean ± SD, LC→RO), and the time interval of each gait event in the walking stage was: 238 ± 15 ms (mean ± SD, RO→RC), 245 ± 18 ms (mean ± SD, RC→LO), 258 ± 16 ms (mean ± SD, LO→LC). For the convenience of comparative analysis, we define the duration of the preparation phase as 750 ms, and the interval between gait events in the walking phase was uniformly defined as 250 ms.

### Data acquisition

EEG signals were recorded by a home-designed 32-channel flexible electrode array and Plexon OmniPlex system (Plexon Inc., Dallas, TX, USA). Figure 1A and B shows the actual electrode image and the position of the electrode on the rat skull. The electrode array contains 34 channels, including 32 working channels, a reference channel (REF), and a ground channel (GND). It was worth noting that the signals from the REF and GND channels were excluded, and only the signals from the 32 working channels were used for subsequent analysis. The electrode array was connected to the Digital Headstage processor (Plexon Inc., Dallas, TX, USA) through a cable, and the EEG signal was sampled at 2,000 Hz for storage.

### EEG data analysis

#### EEG data preprocessing

**Fig. 2.**
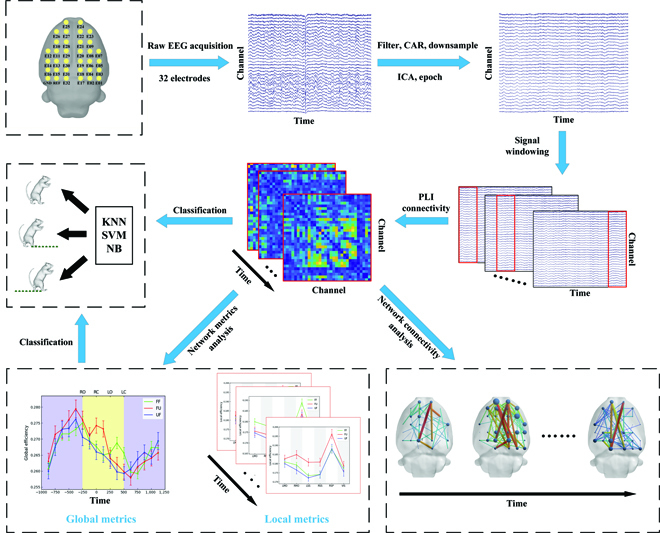
EEG data processing procedure. CAR, common average; ICA, independent component analysis; VIS, visual area; RSS, right somatosensory area.

Figure 2 shows the processing procedure for EEG data. First, the raw EEG signals from the 32-channel electrode array were notch filtered at 50 Hz to reduce line noise. Our previous study showed that cortical activity in rats was concentrated in the 3- to 50-Hz frequency band when encountering unexpected terrain [19], so the signal was filtered at 3 to 50 Hz. Next, The EEG data were re-referenced to the common average and down-sampled to 500 Hz. Subsequently, the EEG data were decomposed into independent component (IC) sources by an independent component analysis algorithm. We removed those non-brain IC sources by visually inspecting each IC scalp projection and power spectrum to obtain clean EEG data. After the above processing, we extracted epoch from the EEG data of each rat according to the mean hind limb kinematics data of all rats (each epoch started 1 s before the event RC and the total duration was 2.5 s, including 2 complete preparation phases and 1 complete walking phase). After that, epochs from all rats were divided into 3 groups according to different task conditions (FF: 685 epochs, FU: 586 epochs, and UF: 575 epochs). Each rat contributed a similar number of epochs under each condition). The above EEG data analysis was performed using custom scripts written in MATLAB v2018b (The MathWorks, Inc., Nedick, MA, USA) containing functions from EEGLAB [20].

#### Functional connectivity computation

Each electrode node's EEG signal reflects the neurons' activity in a specific range of brain areas below the node [21]. The functional connectivity can be used to assess the functional relationship between brain regions, quantified with measures of statistical dependencies [22]. Phase synchronization was an effective measurement of functional connectivity. PLI (a phase synchronization-based functional connectivity index) was widely used in EEG signal correlation analysis due to its robustness to the presence of common sources [23]. Therefore, the PLI between the signals of each electrode node was calculated to evaluate the functional relationship among different brain areas. For 2 given EEG signals ***x***, ***y***, PLI values can be calculated by the following equations:

$${\mathrm{PLI}}_{\mathrm{xy}}=\left|\frac{1}{N}\sum_{n=1}^{N}\mathrm{sign}\left(\Delta \phi_{\mathrm{xy}}\left(t_{n}\right)\right)\right|$$ (1)

$$\Delta \phi_{\mathrm{xy}}\left(t_{n}\right)=\arctan\frac{x_{H}\left(t_{n}\right)}{x\left(t_{n}\right)}-\arctan\frac{y_{H}\left(t_{n}\right)}{y\left(t_{n}\right)}$$ (2)

The PLI value between 0 and 1: 0≤𝑃𝐿𝐼≤1, where 0 indicates no coupling or coupling with a phase difference cantered around 0 mod, and 1 indicates completed phase locking at a value of Δ*ϕ_xy_*(*t_n_*) different from 0 mod. In Eq. 1, | ***X*** | represents the absolute value of X, where ***sign***( ) was a signum function. If ***X*** > 0, ***sign***(X) = 1; if ***X*** < 0, ***sign***(***X***) = −1; if ***X*** = 0, ***sign***(***X***) = 0. In Eq. 1, Δ*ϕ_xy_*(*t_n_*) denotes the instantaneous phase difference between signals x and y, which can be computed by Eq. 2. where *x_H_*(*t_n_*) and *y_H_*(*t_n_*) were the Hilbert transforms of *x*(*t_n_*) and *y*(*t_n_*), respectively. In this study, we based on the duration of the epoch and gait phase to select a 250-ms long time window to scan the EEG signal. At the same time, there was a 50% overlap between the windows for a more continuous change in connectivity. The HERMES toolbox [23] was used to calculate the signals' PLI [24] matrix within each time window. Next, we got *N* × *T* × *CH* × *CH* (1,846 × 19 × 32 × 32) PLI matrices, where *N* was the number of epochs, *T* was the number of time windows, and *CH* was the number of electrode nodes. It is worth noting that the preparation phase was a gait phase with a long duration (from −1,000 to −250 ms, i.e, 750 ms before event RO), and using a 250-ms time window (50% overlap) scan resulted in 5 PLI matrices. To facilitate comparative analysis with other gait phases, we averaged the first, third, and fifth PLI matrices to obtain the mean PLI matrix (1,846 × 1 × 32 × 32) of the whole preparation phase.

#### Functional network construction

We constructed the functional network of the rat brain on the spatial and temporal scales by using the spatial position of each electrode point on the rat skull and the time-varying PLI matrix. In order to build networks of different gait phases, we extracted the matrix belonging to the walking phase from the PLI matrix calculated before and merged them to get a final size of 1,846 × 4 × 32 × 32 PLI matrix. Afterward, we averaged the PLI matrices within each gait phase from the same conditions, resulting in the averaged PLI matrices under the 3 experimental conditions (matrix size was 3 × 4 × 32 × 32, where 3 represents three task conditions, 4 represents four gait phases, and 32 represents different electrode channels). After obtaining the average adjacency matrix for each gait phase under different task conditions, we arranged all electrode points by spatial location (Fig. 1C). Then, by querying the PLI matrix, it was determined whether each electrode point was connected to other electrode points and the weight of the connection. We connect the connected electrode points with lines and use the thickness and color of the lines to indicate the strength of the connection. Additionally, the size of each electrode point was used to indicate the number of connections the electrode point has. It was worth mentioning that the edge threshold of the network was set to 50% of the strongest connections (i.e., only keep edges that are more than 50% of the strongest connections) [25]. Finally, the BrainNet Viewer toolbox [26] was used to visualize the brain functional network.

#### Network metrics analysis

In this study, the Brain Connectivity Toolbox (http://www.nitrc.org/projects/bct/) was used to calculate network metrics. First, the mean global and local metrics of the functional network of all rats were calculated to analyze the changes in the functional connectivity of the rat brain over time under different task conditions. For the global metrics, we calculate the global efficiency, transitivity, and characteristic path length of the whole brain function network in each time window and average the global metrics belonging to the same condition. In addition, we also calculate the global metrics belonging to each gait phase according to the PLI matrix of each gait phase. In particular, the global metrics of the preparation phase were calculated on the basis of the average PLI matrix of the preparation phase (LC→RO). These global metrics measure the brain's ability to combine information from different brain regions rapidly. Global efficiency was an index to measure the efficiency of distant information transmission in the network. It was defined as the inverse of the average characteristic path length between all nodes in the network [27]. The characteristic path length was the average shortest path length between all node pairs in the network, indicating how easily information can be transferred across the network [28]. The transitivity of the network reflects the prevalence of clustered connectivity around individual nodes [29]. For local metrics, the local efficiency [30], node strength [31], clustering coefficient [32], and eigenvector centrality [33] of each node in each gait phase were calculated. Similar to global metrics, the local metrics of the preparation phase were also calculated on the basis of the average PLI matrix of the preparation phase (LC→RO). The local efficiency was an index to measure the information transfer efficiency of each node in the network. The strength of the relationship between the individual node and other nodes in the network was called node strength, which can be computed as the sum of the connectivity weights of the edges attached to each node. The clustering coefficient was the fraction of a node's neighbors that were neighbors of each other, and eigenvector centrality was a measure of the node's influence on a network. Then, we averaged the local metrics of all nodes belonging to the same brain area and the same condition to obtained the average local metrics of each brain area. It is worth mentioning that the brain region to which each electrode point belongs (Fig. 1C) was determined according to the stereotaxic coordinates of the rat brain [34], the size of the electrode (Fig. 1A), and the installation position of the electrode on the rat skull (Fig. 1B). Finally, IBM SPSS Statistics for Windows, version 27.0 (IBMCorp., Armonk, NY, USA) was used to perform a 1-way analysis of variance for the significance (*P* < 0.05) of the network metrics across different task conditions.

#### Machine learning algorithms for the classifying locomotion states

This study used 3 classifiers, SVM, NB, and KNN, for locomotion state detection. These classification algorithms were all implemented by the Scikit-Learn toolbox based on python v3.8 [35]. The basic principle of SVM was to find a hyperplane in the sample space to separate the samples of different classes and to maximize the minimum distance between the classified points and this plane [36]. The NB classifier was a statistical classifier based on Bayes' theorem, which can predict class membership probabilities [37]. The basic idea of KNN was to judge which category the sample belongs to according to the category of the K points closest to the sample [38].

In order to compare the difference between time-varying network analysis method and other analysis methods in detecting rat locomotion state, we used raw EEG signal, whole-time PLI matrix, and time-varying PLI matrix in a complete gait cycle (i.e., LC→RO→RC→LO→LC) as features to classify rat locomotion state. The time length of the raw EEG signal was 1.5 s (i.e., −1,000 to 500 ms). Since the sampling rate was 500 Hz, the matrix of the EEG signal is *N* × 32 × 750 (FF: *N* = 685, FU: *N* = 586, UF: *N* = 575, input feature: 32 × 750 = 24,000). The whole-time PLI matrix was calculated from the EEG signal within −1,000 to 500 ms, and the size was *N* × 32 × 32 (input feature dimension: 32 × 32 = 1,024). For the time-varying PLI, a matrix of size *N* × 4 × 32 × 32 (4 represents four gait phases, i.e., preparation phase: LC→RO; walking phase: RO→RC, RC→LO, and LO→LC) was obtained, and the input feature dimension was 4,096 (4 × 32 × 32 = 4,096). Then, we use *t* test to analyze the significance of the classification results of each classifier. Furthermore, we use the global and local metrics of the time-varying network for all gait phases (4 phases in total) as features to classify the locomotion state of rats. For the global metrics (global efficiency, transitivity, and characteristic path length), a matrix of size *N* × 4 × 1 × 1 was obtained, and the input feature dimension was 4. Moreover, for the local metrics (local efficiency, node strength, clustering coefficient, and eigenvector centrality), we get a matrix of size *N* × 4 × 32 × 1, and the input feature dimension was 128. Finally, the effect of the gait phase on the classification performance was also evaluated. First, we extract the PLI matrix of each gait phase (i.e., LC→RO, RO→RC, RC→LO, and LO→LC). The size of the PLI matrix for a single gait phase was *N* × 1 × 32 × 32, and the input dimension was 1,024. Next, we merged PLI matrices of different gait phases according to the sequence of gait events (i.e., LC→RO→RC, LC→RO→RC→LO, and LC→RO→RC→LO→LC). The merged PLI matrix sizes were *N* × 2 × 32 × 32, *N* × 3 × 32 × 32, and *N* × 4 × 32 × 32, and the input feature dimensions were: 2,048, 3,072, and 4,096, respectively. It is worth mentioning that we did not train classifiers to separate all these gait phases but manually extracted data belonging to different gait phases. Finally, we sequentially use the PLI matrix of a single gait phase and the combined PLI matrices of multiple gait phases as features to detect the locomotion state of rat.

In this study, we obtained the average of classification results through 5-fold cross-validation. Specifically, all the datasets were divided into 5 parts, 1 of which was taken as the test set without repetition, and the other 4 were used as the training set to train the model. After that, the classification results of the model on the test set were calculated each time, and all classification results were averaged to obtain the final classification results. In order to evaluate the performance of the classifier more comprehensively, we use the accuracy and F1 score as evaluation metrics. Accuracy was defined as the proportion of correctly classified samples to the total samples, while F1 score was the harmonic mean of precision and sensitivity. Precision was the proportion of true positives (TP) to the sum of TP and false positives (FP), and sensitivity was defined as the proportion of the TP to the sum of the TP and false negatives (FN) [39].

## Results

### Dynamic brain functional networks in rats

**Fig. 3.**
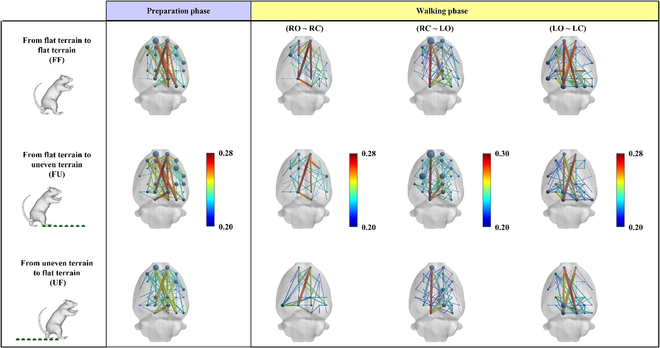
Time-varying brain function networks. Each row represents a time-varying brain functional network under a specific task condition (FF, FU, and UF). Each column represents a locomotion phase. The node size denotes the degree, and the color and size of the edges represent the connection weight. Connections below 50% of the strongest connection were removed.

Figure 3 shows the network connections under 3 different task conditions for each locomotion phase. We noticed that connections with big weights (red line) in the preparation phase appeared in the left somatomotor area (LMO), right somatomotor area (RMO), and retrosplenial area (RSP), while the strongest connection occurred between RMO and RSP. It was worth noting that the edges of the right somatosensory area were more than the left. In addition, compared with other phases under the same task condition, the connectivity of the preparation phase was stronger (larger edge weight and greater degrees). Interestingly, during the right hind limb swing (RO→RC) after the rats finished the preparation phase, under all task conditions, connections between RMO and RSP also remained high level. However, the connection weights between LMO and RSP were weakened compared to the preparation phase and much smaller than the connection between RMO and RSP. In addition, compared with other conditions, the left somatosensory area (LSS) under the UF condition has more edges, and the weight of the edges was relatively larger. Interestingly, the degree and weight of LSS under FU and UF conditions were larger than those under FF condition in the RC→LO phase and more evident in the FU condition. At the end of the walking phase, during the swing of the rat's left hind limb (LO→LC), most connections appeared between the RSP and the LSS. It was worth noting that the largest connection weight appears between RMO and RSP in the FU condition, while the opposite was confirmed in the FF and UF conditions. Furthermore, the degree and connection weight of LSS under FF and FU conditions were larger than those under UF condition. The connection weights under the UF condition were smaller than those in the other conditions, which occurs in all gait phases.

### Brain functional network properties

#### Dynamic global metrics of network

**Fig. 4.**
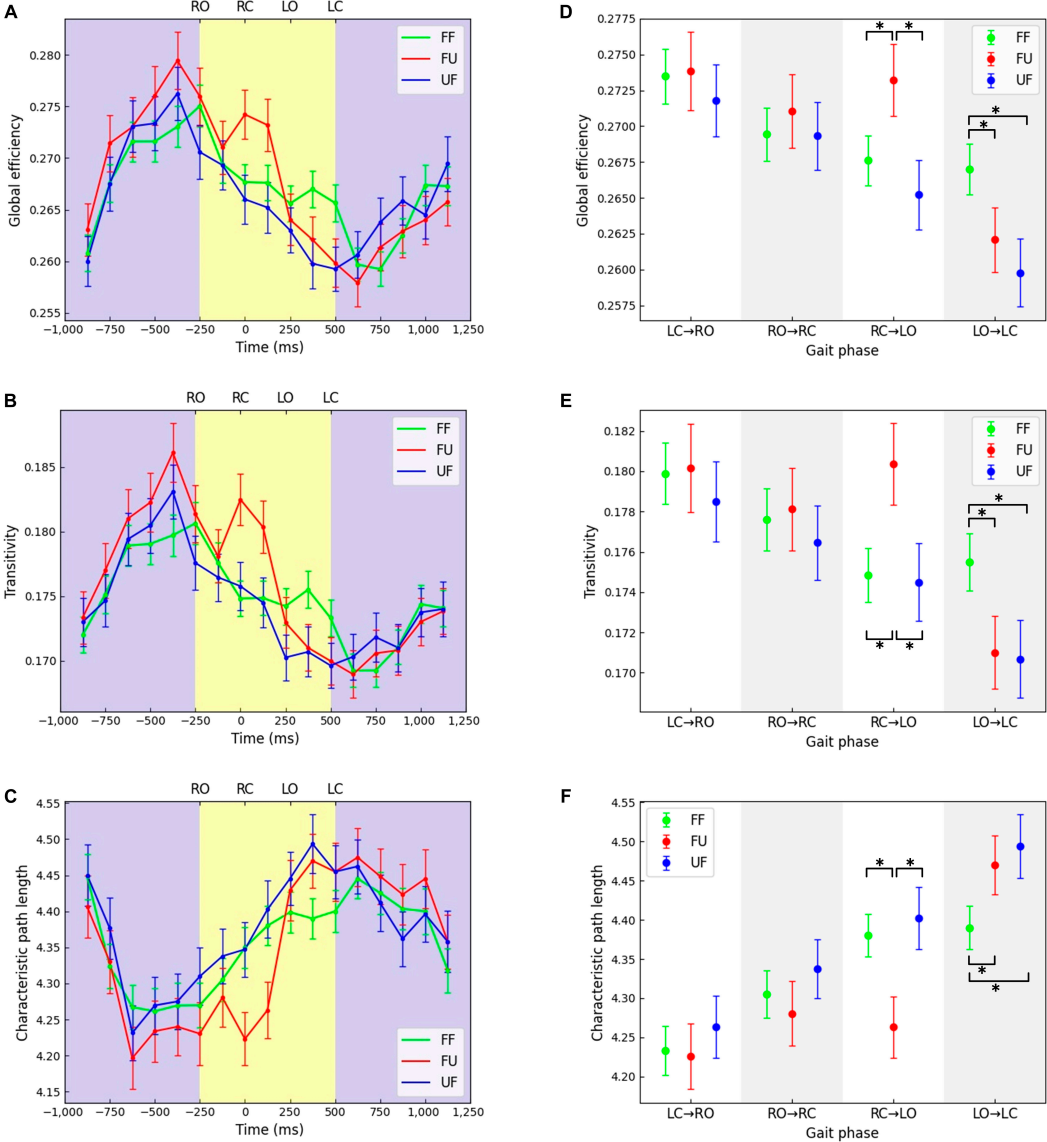
Time-varying global metrics of the brain network. The curve of (A) global efficiency, (B) transitivity, and (C) characteristic path length with time. In each subfigure, the green, red, and blue lines indicate the corresponding network properties of the FF, FU, and UF conditions, respectively. The yellow window denotes the walking phase, and the purple window denotes the preparation phase. (D) Global efficiency, (E) transitivity, and (F) characteristic path length of different gait phases. All error bars indicate the 95% confidence interval of the mean. *P < 0.05.

Figure 4 shows the time-varying global metrics of the functional network. We note that in the first preparation phase (purple window), global efficiency and transitivity (Fig. 4A and B) keep increasing until the end of the preparation phase. In contrast, during the walking phase (yellow window), both decrease until the walking phase ends, and they gradually recovered to the same level as the first preparation phase in the second preparation stage. The characteristic path length shows the opposite trend to other global metrics (Fig. 4C). Interestingly, in a period of time (RC→LO) after the right hind paw contacted the uneven terrain (FU condition), both the global efficiency (Fig. 4D) and the transitivity (Fig. 4E) were significantly improved compared with the FF and UF conditions, while the characteristic path length (Fig. 4F) was significantly decreased (global efficiency: *F*(2,1782) = 3.185, *P* = 0.041; post hoc tests: FF vs. FU: *P* = 0.038, FF vs. UF: *P* = 0.738, FU vs. UF: *P* = 0.022; transitivity: *F*(2,1766) = 3.247, *P* = 0.039; post hoc tests: FF vs. FU: *P* = 0.024, FF vs. UF: *P* = 0.876, FU vs. UF: *P* = 0.037; characteristic path length: *F*(2,1827) = 4.035, *P* = 0.018; post hoc tests: FF vs. FU: *P* = 0.008, FF vs. UF: *P* = 0.961, FU vs. UF: *P* = 0.019). Then, during the left hind limb swing (LO→LC), the global efficiency (Fig. 4D) and transitivity (Fig. 4E) were significantly reduced in the FU and UF conditions compared to the FF condition, while the characteristic path was significantly increased (Fig. 4F) (global efficiency: *F*(2,1795) = 5.585, *P* = 0.004; post hoc tests: FF vs. FU: *P* = 0.021, FF vs. UF: *P* = 0.002, FU vs. UF: *P* = 0.481; transitivity: *F*(2,1793) = 4.103, *P* = 0.017; post hoc tests: FF vs. FU: *P* = 0.038, FF vs. UF: *P* = 0.011, FU vs. UF: *P* = 0.631; characteristic path length: *F*(2,1819) = 5.138, *P* = 0.006; post hoc tests: FF vs. FU: *P* = 0.014, FF vs. UF: *P* = 0.001, FU vs. UF: *P* = 0.435).

#### Brain network local metrics

**Fig. 5.**
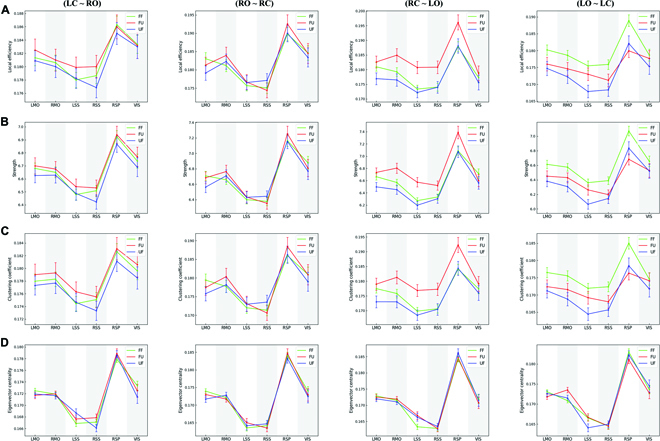
Brain network local metrics of each brain region in different gait phases. (A) Local efficiency. (B) Strength. (C) Clustering coefficient. (D) Eigenvector centrality. Each column represents a gait phase. In each subfigure, the green, red, and blue lines indicate the corresponding network properties of the FF, FU, and UF conditions, respectively. All error bars indicate the 95% confidence interval of the mean.

Figure 5 shows the local metrics of each brain region in all gait phases. Similar to the global metrics, the local metrics of multiple brain regions in the RC→LO and LO→LC phases showed obvious difference under 3 different task conditions. However, there was no significant difference in the local metrics of each brain region under the 3 task conditions of LC→RO phase (preparation stage) and RO→RC phase. Specifically, in the RC→LO phase, the local metrics of almost all brain regions under the FU condition were stronger than those under other conditions, while in the LO→LC phase, the local metrics under the FF condition were stronger than those under FU and FU conditions. It was worth noting that in the RC→LO phase, the values of local efficiency (Fig. 5A), node strength (Fig. 5B), and clustering coefficient (Fig. 5C) of LMO under UF condition were smaller than those under other conditions. In addition, in the LO→LC phase, the value of the eigenvector centrality of the RMO under the FU condition was larger than that in the other conditions. Furthermore, in the RC→LO phase, the value of eigenvector centrality (Fig. 5D) of LSS under FU and UF conditions was larger than that under FF condition. In the LO→LC phase, the values of all local metrics of LSS under UF condition were smaller than those under FF and UF conditions. Moreover, all local metrics of RSP maintained the highest level in all task conditions, higher than other brain regions under the same condition. All local metrics of the visual area were very close under all conditions.

### Analysis of classification results

**Fig. 6.**
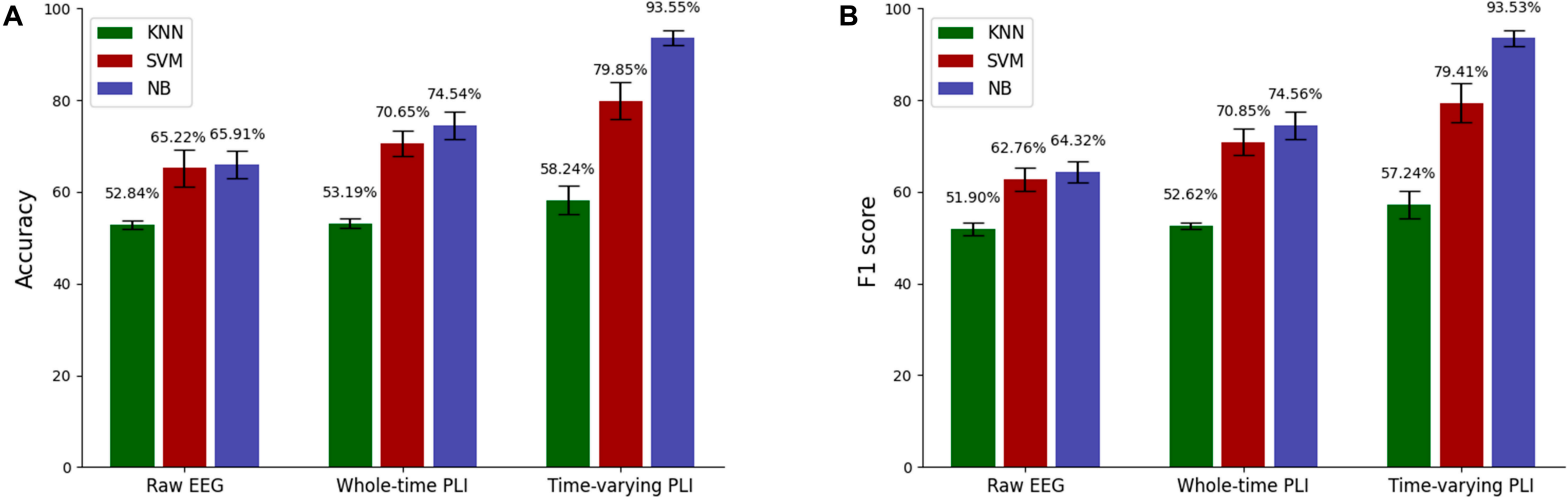
(A) Accuracy and (B) F1 score of different classifiers when the raw EEG signal, whole time PLI, and time-varying PLI were used as features, respectively. In each subfigure, the green, red, and blue bars indicate the performance of the KNN classifier, SVM classifier, and NB classifier, respectively. All error bars indicate the 1 SD of the mean.

**Fig. 7.**
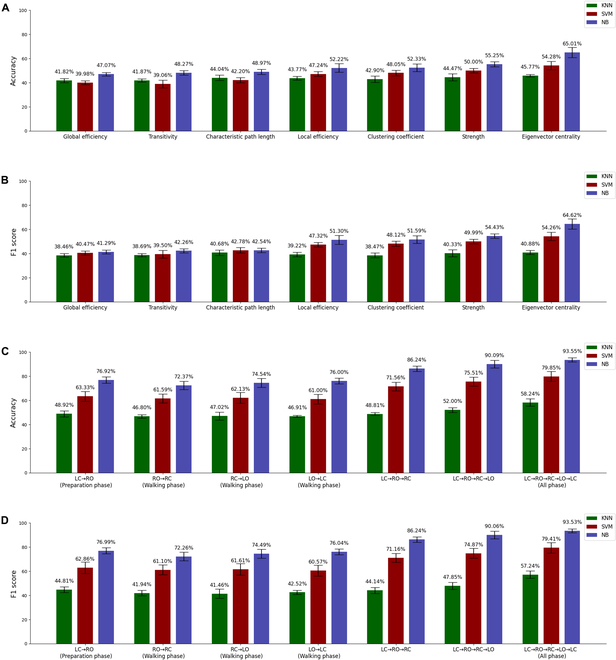
(A) Accuracy and (B) F1 score of different classifiers in classifying brain network metrics (global efficiency, transitivity, characteristic path length, local efficiency, strength, clustering coefficient, and eigenvector centrality). (C) Accuracy and (D) F1 score of classifiers in classifying PLI matrix of different gait phases. In each subfigure, the green, red, and blue bars indicate the performance of the KNN classifier, SVM classifier, and NB classifier, respectively. All error bars indicate the 1 SD of the mean.

Figure 6 shows the classification accuracy and F1 score of the 3 classifiers when using the raw EEG signal, whole-time PLI, and time-varying PLI as features to classify rat locomotion states. Clearly, all classifiers perform significantly better (*P* < 0.05) when using time-varying PLI as input features compared to using raw EEG signals and whole-time PLI. In addition, when using time-varying PLI as a feature for classification, the accuracy and F1 score of the NB classifier exceeded 95%, followed by the SVM classifier (about 79%), and the KNN classifier performs the worst (about 58%). Figure 7A and B shows the accuracy and F1 score of the classifier when using time-varying network global and local metrics as features. In general, among all the metrics, the classification result of the Eigenvector centrality was the best. Among the 3 classifiers, NB has the best performance, followed by SVM, and the worst performance was KNN (accuracy and F1 score for all metrics were below 50%). The largest accuracy and F1 score come from the classification of the Eigenvector centrality by NB, which were 65.01% and 65.42%, respectively. Figure 7C and D shows the classification accuracy and F1 score of the 3 classifiers for the PLI matrices of individual and combined gait phases. Similarly, NB still performs best among the 3 classifiers, and KNN still performs worst. We note that for the same classifier, there was little difference in classification performance (the accuracy and F1 score of NB were around 75%, the accuracy and F1 score of SVM were about 60%, and the accuracy and F1 score of KNN were about 45%) when the PLI matrix of a single gait phase (preparation phase, RO→RC, RC→LO and LO→LC) was used as the input feature. However, as the number of PLI matrices from different gait phases increases (LC→RO→RC, LC→RO→RC→LO, and LC→RO→RC→LO→LC), the accuracy and F1 score of all classifiers show an upward trend (the accuracy and F1 score of NB and SVM increased significantly, while the accuracy and F1 score of KNN increased slightly) and reach a maximum after merging the PLI matrices of all gait phases.

## Discussion

In this study, we analyzed the time-varying functional connectivity of rats when encountering unexpected terrain and proposed a method for classifying rat locomotion states based on time-varying network properties. Our results show that the brain functional connectivity of the rat varied with gait phases and terrain conditions. Furthermore, the classification results show that combining the PLI matrices of all gait phases as classification features achieves better classification performance, and the NB classifier exhibited the best performance. Our findings have important implications for understanding animals’ motor control mechanisms in adapting to unexpected terrain.

Our results show that the brain functional connectivity varied with gait phases. Overall, the connectivity in the preparation phase was stronger than in the walking phase. Specifically, the degree and connection weights in the preparation phase were larger than those in the walking phase, and the global efficiency and transitivity reach the highest at the end of the preparation stage and gradually decrease in the walking stage, while the characteristic path length shows the opposite trend (Figs. 3 and 4). A previous study of human walking showed that brain functional connectivity during walking phase was weaker than during standing phase [40]. From our results, this rule also applies to rat bipedal walking on a treadmill and does not change with different terrain. It is worth noting that the degree of participation of the cortex was related not only to the complexity of the task but also to the degree of volitional engagement [41,42]. During the preparation phase, the rat stands on the treadmill in a bipedal position, and the rat's body begins to tilt as the treadmill belt turns. The gradual increase of the tilt angle seriously disrupted the body balance of the rats, which gives the rat a strong desire to adjust the body. In this process, high-level motor function was recruited, which makes the information interaction between different cortical areas stronger. A study of rat walking suggests that the details of hind limb motor control in rats were completed by the spinal cord [43]. We speculate that the decrease of cortical functional connectivity in rats during walking phase was probably due to the fact that the cortex enters the idling state after releasing the motor task in the preparation phase, while the spinal controls the movement according to the task during walking. Interestingly, we noticed that the cortex seemed to be more concerned with the regulation of the hind limb that support the body. We found that in the double-support phase (preparation phase and RC→LO), high-level connectivity appeared in the bilateral somatomotor areas. In the single-support phase (RO→RC and LO→LC), the connectivity of the somatomotor area on the opposite side of the supporting hind limb was much stronger than that on the other side. Similar to our results, a previous study showed that the motor cortex was more interested in stance control than the hind limb swing [43]. This may be related to the higher demand for muscle activity of the hind limb supporting the body, and the somatomotor area contributes directly to changes in muscle activity [44]. In the double-support phase, the weight of the body was shared by both hind limb, and the muscle strength required by both hind limb was similar, resulting in the similar connectivity level of the bilateral somatomotor areas. In the single-support phase, the weight of the body was completely borne by 1 hind limb, which requires not only stronger muscle strength but also different muscles to cooperate with each other to maintain stable posture. It is worth noting that the somatosensory cortex also shows a similar pattern, and the change of the somatosensory cortex was probably caused by the change of gait. In general, bipedal animals gradually transfer their body weight to the opposite side of the swinging hind limb in the double-support phase, and in the swing phase, the body weight was supported completely by 1 hind limb [45–47]. This leads to increased muscle strength from supporting hind limb, which leads to an increase in sensory information coming from the muscles. The transition from 2-foot support to single-foot support increases the pressure on the tactile receptors of the paw, which also increases the incoming sensory information. The increase in incoming sensory information makes the somatosensory area more active in interacting with other cortical areas so that the brain can generate appropriate control instructions. Notably, RSP was recruited at all gait phases and connected to all cortical areas, and eigenvector centrality (Fig. 5D) also indicates its importance in adapting rats to unexpected terrain. This may be related to the functions of RSP with multisensory information integration, terrain feature information recognition, and motion planning [48–50].

Previous studies had shown that the cerebral cortex was more active when humans encountered unexpected external disturbances [5–7]. We also found that the brain information transmission ability increased in a short time after the rat's accidental contact with uneven terrain (RC→LO), manifested as a significant increase in global efficiency and transitivity and a significant decrease in characteristic path length (Fig. 4). In addition, (Fig. 5A to C) shows that local metrics of multiple brain regions were significantly increased under FU condition during this process. Obviously, this difference comes from uneven terrain, which makes the incoming sensory information different. In addition, the input of sensory information was crucial for gait adjustment in unpredictable environments, and the sensory cortex undoubtedly plays an important role in the adaptation of animals to unfamiliar environments [51,52]. In this study, the functional connectivity of the somatosensory area changed obviously. Specifically, after the rats were accidentally in contact with uneven terrain (FU), the degree and edge weights of the LSS were far greater than in contact with flat terrain (Fig. 3). Changes in eigenvector centrality also indicated the importance of the somatosensory cortex in rat adaptation to terrain changes (Fig. 5D). We noticed that the eigenvector centrality of LSS was much greater than that of walking on flat terrain (FF) after rats accidentally contact with uneven terrain (FU). The possible reason for this phenomenon was that, compared with the flat surface, the pressure on the tactile receptor of rat paws after contacting the uneven surface increases, thus more tactile information was transmitted. In addition, due to the uneven support surface, the state of the hind limb muscles of rats has changed obviously compared with that before, which changes the sensory information transmitted by muscles. These sensory information from muscles and skin play an important role in the motor recovery and gait update after sudden disturbance [53–56]. In this process, the somatosensory cortex not only needs to process a large amount of sensory information in a short time but also needs to interaction with other cortical regions as soon as possible to help complete motion planning in a timely manner. Obviously, this puts forward higher requirements for the computing ability of the cortex and consumes more energy. Interestingly, compared to normal walking (FF), there was a obvious reduction in brain connectivity during the transition between different terrains (FU) by swinging the hind limb (LO→LC). This was reflected in significant decreases in global efficiency and transitivity and increases in characteristic path length, as well as significant reductions in local metrics across multiple brain regions (Fig. 5A to C). Previous studies have shown that the spinal play an essential role in coping with terrain change [53,56]. Therefore, in order to enable the brain to recover the consumption caused by the previous fast calculation as soon as possible, the motion control during terrain transition may rely more on the spinal than normal walking. To our surprise, the connectivity under UF conditions was much weaker than that under FF and FU conditions, as seen in Fig. 3. In addition, the local metrics of LMO (Fig. 5A to C) and LSS (Fig. 5) were much smaller in the UF condition than in the other 2 conditions. In the swing phase, the global metrics were also significantly lower than that of normal walking (Fig. 4). As the uneven area used in this study was only 11 × 11 cm in size, unable to support continuous walking of rats. Therefore, after the rat completely transits to the uneven terrain, the next step must leave the uneven terrain and enter the flat terrain, which enables the rat to predict the terrain ahead when leaving the uneven terrain. Compared with the unpredictable situation, rats rely more on the spinal to regulate movement when they can predict environmental changes [51,57]. We speculate that in the face of a predictable environment, the cortex was less involved to reduce consumption and relies on spinal regulation to complete the transition of the terrain.

Some studies have shown that using raw EEG signals or whole-time functional network metrics as features can effectively detect the locomotion state of subjects [25,58]. Our results (Fig. 6) show that using the PLI of the time-varying network as a feature was more effective in detecting the locomotion state of rats during adaptation to unexpected terrain than using raw EEG signals or whole-time functional network metrics. This shows that compared with the other 2 methods, using time-varying functional network to analyze the process of rats adapting to unexpected terrain can obtain more useful information. The classification results (Fig. 7) show that using the time-varying adjacency matrix of the network as a feature classification works best among all network metrics. In addition, the classification results of PLI matrices for different gait phases (Fig. 7C and D) show that classification using PLI matrices of multiple gait phases combined in chronological order leads to higher accuracy than classification using PLI matrices of a single gait phase. A previous study showed that the classification accuracy tends to increase as the number of available features increases and remains constant after the number of features reaches a certain number [59]. Obviously, the improvement of classifier performance was directly related to the number of features, and the PLI matrix synthesizes the global and local features of the functional network, so that the classifier can extract more useful features for classification. In addition, our analysis shows that functional connectivity varies with gait phases, which makes each gait phase possess features that were different from other gait phases. Therefore, combining network metrics for multiple gait phases yields more useful features than using a single gait phase network metric.

There were also some limitations of this study. In this study, we did not consider the effect of rat learning on functional connectivity. However, considering the random occurrence of uneven areas, and the change of position and angle of contact between rat paws and uneven areas each time, we believe that the learning of rats was not enough to affect the experimental results. In the future work, we can further discuss the relationship between functional connectivity and the number and the order of trials to clarify the impact of rat learning on functional connectivity. In addition, we only analyzed functional connectivity within a wide frequency range, while analyzing functional connectivity in different frequency bands may get more information. Finally, this study only focuses on the influence of terrain changes caused by the accidental appearance of uneven terrain on the functional connectivity but does not analyze the impact of other kinds of terrain. This may hamper the possibility of generalizing the current results, so it will be necessary to add more types of terrain to verify the current results in the future work. It is worth mentioning that discussing the individual differences in adapting to unexpected terrain was also an important future work for us.

In the present study, we performed a time-varying analysis of functional connectivity in bipedal walking rats that encountered unexpected terrain. Dynamic functional connectivity maps, time-varying functional network global metrics, and local network metrics for different gait phases suggest that information interactions between cortical areas vary with gait phase and terrain conditions. In addition, the classification results emphasize the potential of EEG-based dynamic functional network analysis in animal locomotion detection. The findings of this study deepen our understanding of the role of the cortex in the adaptation of animals to unexpected terrain from the network viewpoint.

## Data Availability

The datasets obtained during the current study are available from the corresponding author on reasonable request.

## References

[1] Kent JA, Sommerfeld JH, Mukherjee M, Takahashi KZ, Stergiou N. Locomotor patterns change over time during walking on an uneven surface. J Exp Biol. 2019;222(Pt 14):jeb202093. 31253712 10.1242/jeb.202093 PMC6679350

[2] Pijnappels M, Bobbert MF, van Dieën JH. Contribution of the support limb in control of angular momentum after tripping. J Biomech*.* 2004;37(12):1811–1818. 15519588 10.1016/j.jbiomech.2004.02.038

[3] Marigold DS, Patla AE. Strategies for dynamic stability during locomotion on a slippery surface: Effects of prior experience and knowledge. J Neurophysiol. 2002;88(1):339–353. 12091559 10.1152/jn.00691.2001

[4] Dusane S, Bhatt T. Can prior exposure to repeated non-paretic slips improve reactive responses on novel paretic slips among people with chronic stroke? Exp Brain Res. 2022;240(4):1069–1080. 35106605 10.1007/s00221-021-06300-8 PMC9290783

[5] Nordin AD, Hairston WD, Ferris DP. Human electrocortical dynamics while stepping over obstacles. Sci Rep. 2019;9(1):4693. 30886202 10.1038/s41598-019-41131-2 PMC6423113

[6] Sipp AR, Gwin JT, Makeig S, Ferris DP. Loss of balance during balance beam walking elicits a multifocal theta band electrocortical response. J Neurophysiol. 2013;110(9):2050–2060. 23926037 10.1152/jn.00744.2012 PMC3841925

[7] Solis-escalante T, Van Der Cruijsen J, De Kam D. Cortical dynamics during preparation and execution of reactive balance responses with distinct postural demands. NeuroImage. 2019;188:557–571. 30590120 10.1016/j.neuroimage.2018.12.045

[8] Beloozerova IN, Sirota MG, Swadlow HA, Orlovsky GN, Popova LB, Deliagina TG. Activity of different classes of neurons of the motor cortex during postural corrections. J Neurosci. 2003;23(21):7844–7853. 12944514 10.1523/JNEUROSCI.23-21-07844.2003 PMC6740594

[9] Beloozerova IN, Sirota MG, Orlovsky GN, Deliagina TG. Activity of pyramidal tract neurons in the cat during postural corrections. J Neurophysiol. 2005;93(4):1831–1844. 15525811 10.1152/jn.00577.2004

[10] Astolfi L, Cincotti F, Mattia D, Marciani MG, Baccala LA, de Vico Fallani F, Salinari S, Ursino M, Zavaglia M, Ding L, et al. Comparison of different cortical connectivity estimators for high-resolution EEG recordings. Hum Brain Mapp. 2007;28(2):143–157. 16761264 10.1002/hbm.20263 PMC6871398

[11] Zhang F, Daducci A, He Y, Schiavi S, Seguin C, Smith RE, Yeh C-H, Zhao T, O'Donnell LJ. Quantitative mapping of the brain’s structural connectivity using diffusion MRI tractography: A review. NeuroImage. 2022;249:118870. 34979249 10.1016/j.neuroimage.2021.118870 PMC9257891

[12] Rossini PM, di Iorio R, Bentivoglio M, Bertini G, Ferreri F, Gerloff C, Ilmoniemi RJ, Miraglia F, Nitsche MA, Pestilli F, et al. Methods for analysis of brain connectivity: An IFCN-sponsored review. Clin Neurophysiol. 2019;130(10):1833–1858. 31401492 10.1016/j.clinph.2019.06.006

[13] Katmah R, Al-Shargie F, Tariq U, Babiloni F, Al-Mughairbi F, Al-Nashash H. A review on mental stress assessment methods using eeg signals. Sensors. 2021;21(15):5043. 34372280 10.3390/s21155043 PMC8347831

[14] Fingelkurts AA, Fingelkurts AA, Kähkönen S. Functional connectivity in the brain — Is it an elusive concept? Neurosci Biobehav Rev. 2005;28(8):827–836. 15642624 10.1016/j.neubiorev.2004.10.009

[15] Silva LM, Stergiou N. The basics of gait analysis. In: Stergiou N, editor. *Biomechanics and gait analysis*. Washington (USA): Academic Press; 2020. p. 225–250.

[16] Gwin JT, Gramann K, Makeig S, Ferris DP. Electrocortical activity is coupled to gait cycle phase during treadmill walking. NeuroImage. 2011;54(2):1289–1296. 20832484 10.1016/j.neuroimage.2010.08.066

[17] Li F, Peng W, Jiang Y, Song L, Liao Y, Yi C, Zhang L, Si Y, Zhang T, Wang F, et al. The dynamic brain networks of motor imagery: Time-varying causality analysis of scalp EEG. Int J Neural Syst. 2019;29(1):1850016. 29793372 10.1142/S0129065718500168

[18] Telesford QK, Lynall M-E, Vettel J, Miller MB, Grafton ST, Bassett DS. Detection of functional brain network reconfiguration during task-driven cognitive states. NeuroImage. 2016;142:198–210. 27261162 10.1016/j.neuroimage.2016.05.078 PMC5133201

[19] Liu H, Li B, Zhang M, Dai C, Xi P, Liu Y, Huang Q, He J, Lang Y, Tang R. Unexpected terrain induced changes in cortical activity in bipedal-walking rats. Biology. 2021;11(1):36. 35053035 10.3390/biology11010036 PMC8773320

[20] Delorme A, Makeig S. EEGLAB: An open source toolbox for analysis of single-trial EEG dynamics including independent component analysis. J Neurosci Methods. 2004;134(1):9–21. 15102499 10.1016/j.jneumeth.2003.10.009

[21] Pastor J, Vega-Zelaya L. Normative structure of resting-state EEG in bipolar derivations for daily clinical practice: A pilot study. Brain Sci. 2023;13(2):167. 36831710 10.3390/brainsci13020167 PMC9953767

[22] Friston KJ. Functional and effective connectivity: A review. Brain Connect. 2011;1(1):13–36. 22432952 10.1089/brain.2011.0008

[23] Niso G, Bruña R, Pereda E, Gutiérrez R, Bajo R, Maestú F, del-Pozo F. HERMES: Towards an integrated toolbox to characterize functional and effective brain connectivity. Neuroinformatics. 2013;11(4):405–434. 23812847 10.1007/s12021-013-9186-1

[24] Stam CJ, Nolte G, Daffertshofer A. Phase lag index: Assessment of functional connectivity from multi channel EEG and MEG with diminished bias from common sources. Hum Brain Mapp. 2007;28(11):1178–1193. 17266107 10.1002/hbm.20346 PMC6871367

[25] Li B, Zhang M, Liu Y, Hu D, Zhao J, Tang R, Lang Y, He J. Rat locomotion detection based on brain functional directed connectivity from implanted electroencephalography signals. Brain Sci. 2021;11(3):345. 33803159 10.3390/brainsci11030345 PMC7998315

[26] Xia M, Wang J, He Y. BrainNet viewer: A network visualization tool for human brain Connectomics. PLOS ONE. 2013;8(7):e68910. 23861951 10.1371/journal.pone.0068910 PMC3701683

[27] Stanley ML, Simpson SL, Dagenbach D, Lyday RG, Burdette JH, Laurienti PJ. Changes in brain network efficiency and working memory performance in aging. PLOS ONE. 2015;10(4):e0123950. 25875001 10.1371/journal.pone.0123950 PMC4395305

[28] Paldino MJ, Zhang W, Chu ZD, Golriz F. Metrics of brain network architecture capture the impact of disease in children with epilepsy. NeuroImage Clin. 2017;13:201–208. 28003958 10.1016/j.nicl.2016.12.005 PMC5157798

[29] Rubinov M, Sporns O. Complex network measures of brain connectivity: Uses and interpretations. NeuroImage. 2010;52(3):1059–1069. 19819337 10.1016/j.neuroimage.2009.10.003

[30] Latora V, Marchiori M. Efficient behavior of small-world networks. Phys Rev Lett. 2001;87(19):198701. 11690461 10.1103/PhysRevLett.87.198701

[31] Heathrow L, Roman T. Node degree and strength. In: Fornito A, Zalesky A, Bullmore ET, editors. *Fundamentals of brain network analysis*. San Diego (CA): Academic Press; 2016. p. 115–136.

[32] Liu M, Xu G, Yu H, Wang C, Sun C, Guo L. Effects of transcranial direct current stimulation on EEG power and brain functional network in stroke patients. IEEE Trans Neural Syst Rehabil Eng. 2022;31:335–345. 10.1109/TNSRE.2022.3223116 36395131

[33] Fletcher JMK, Wennekers T. From structure to activity: Using centrality measures to predict neuronal activity. Int J Neural Syst. 2018;28(2):1750013. 28076982 10.1142/S0129065717500137

[34] Paxinos G, Watson C. The rat brain in stereotaxic coordinates. Compact sixth edition ed. *Rat Brain Stereotaxic Coord.*; 2009; vol. 3, no. 2, p. 6.

[35] Pedregosa F, Varoquaux G, Gramfort A, Michel V, Thirion B, Grisel O, Blondel M, Prettenhofer P, Weiss R, Dubourg V, et al. Scikit-learn: Machine learning in python. J Mach Learn Res. 2011;12:2825–2830.

[36] Zhang D. Support vector machine. *Fundamentals of image data mining*. Cham (Switzerland): Springer; 2019. p. 179–205.

[37] Abdulrahman SA, Khalifa W, Roushdy M, Salem ABM. Comparative study for 8 computational intelligence algorithms for human identification. Comput Sci Rev. 2020;36:100237.

[38] Guo B. A new data classification improvement approach based on kernel clustering. J Phys Conf Ser. 2021;2082:012021.

[39] Chicco D. Ten quick tips for machine learning in computational biology. BioData Min. 2017;10:35. 29234465 10.1186/s13040-017-0155-3 PMC5721660

[40] Lau TM, Gwin JT, Ferris DP. Walking reduces sensorimotor network connectivity compared to standing. J Neuroeng Rehabil. 2014;11:14. 24524394 10.1186/1743-0003-11-14 PMC3929753

[41] DiGiovanna J, Dominici N, Friedli L, Rigosa J, Duis S, Kreider J, Beauparlant J, van den Brand R, Schieppati M, Micera S, et al. Engagement of the rat hindlimb motor cortex across natural locomotor behaviors. J Neurosci. 2016;36(40):10440–10455. 27707977 10.1523/JNEUROSCI.4343-15.2016 PMC6705591

[42] Beloozerova IN, Sirota MG. Cortically controlled gait adjustments in the cat. Ann N Y Acad Sci. 1998;860:550–553. 9928360 10.1111/j.1749-6632.1998.tb09101.x

[43] Song W, Ramakrishnan A, Udoekwere UI, Giszter SF. Multiple types of movement-related information encoded in Hindlimb/trunk cortex in rats and potentially available for brain-machine Interface controls. IEEE Trans Biomed Eng. 2009;56(11):2712–2716. 19605313 10.1109/TBME.2009.2026284 PMC2883457

[44] Drew T, Marigold DS. Taking the next step: Cortical contributions to the control of locomotion. Curr Opin Neurobiol. 2015;33:25–33. 25643847 10.1016/j.conb.2015.01.011

[45] Iida H, Yamamuro T. Kinetic analysis of the center of gravity of the human body in normal and pathological gaits. J Biomech. 1987;20(10):987–995. 3693380 10.1016/0021-9290(87)90328-9

[46] Nakajima K, Mori F, Takasu C, Mori M, Matsuyama K, Mori S. Biomechanical constraints in hindlimb joints during the quadrupedal versus bipedal locomotion of M. fuscata. Prog Brain Res. 2004;143:183–190. 14653163 10.1016/S0079-6123(03)43018-5

[47] Xu D, Wang Q. Noninvasive human-prosthesis interfaces for locomotion intent recognition: A review. Cyborg Bionic Syst. 2021;2021:9863761. 36285130 10.34133/2021/9863761 PMC9494705

[48] Hindley EL, Nelson AJD, Aggleton JP, Vann SD. Dysgranular retrosplenial cortex lesions in rats disrupt cross-modal object recognition. Learn Mem. 2014;21(3):171–179. 24554671 10.1101/lm.032516.113 PMC3929849

[49] Spiers HJ, Maguire EA. Thoughts, behaviour, and brain dynamics during navigation in the real world. NeuroImage. 2006;31(4):1826–1840. 16584892 10.1016/j.neuroimage.2006.01.037

[50] Carstensen LC, Alexander AS, Chapman GW, Lee AJ, Hasselmo ME. Neural responses in retrosplenial cortex associated with environmental alterations. iScience. 2021;24(11):103377. 34825142 10.1016/j.isci.2021.103377 PMC8605176

[51] Pearson KG. Role of sensory feedback in the control of stance duration in walking cats. Brain Res Rev. 2008;57(1):222–227. 17761295 10.1016/j.brainresrev.2007.06.014

[52] Wang L, Ma L, Yang J, Wu J. Human somatosensory processing and artificial somatosensation. Cyborg Bionic Syst. 2021;2021:9843259. 36285142 10.34133/2021/9843259 PMC9494715

[53] Shinya M, Fujii S, Oda S. Corrective postural responses evoked by completely unexpected loss of ground support during human walking. Gait Posture. 2009;29(3):483–487. 19128973 10.1016/j.gaitpost.2008.11.009

[54] Van Der Linden MH, Marigold DS, Gabreëls FJM, Duysens J. Muscle reflexes and synergies triggered by an unexpected support surface height during walking. J Neurophysiol. 2007;97(5):3639–3650. 17392408 10.1152/jn.01272.2006

[55] Santuz A, Laflamme OD, Akay T. The brain integrates proprioceptive information to ensure robust locomotion. J Physiol. 2022;600(24):5267–5294. 36271747 10.1113/JP283181

[56] Kim Y, Aoi S, Fujiki S, Danner SM, Markin SN, Ausborn J, Rybak IA, Yanagihara D, Senda K, Tsuchiya K. Contribution of afferent feedback to adaptive Hindlimb walking in cats: A Neuromusculoskeletal modeling study. Front Bioeng Biotechnol. 2022;10:825149. 35464733 10.3389/fbioe.2022.825149 PMC9023865

[57] Beloozerova IN, Sirota MG. The role of the motor cortex in the control of accuracy of locomotor movements in the cat. J Physiol. 1993;461:1–25. 8350259 10.1113/jphysiol.1993.sp019498 PMC1175242

[58] Fu Y, Zhou Z, Gong A, Qian Q, Su L, Zhao L. Decoding of motor coordination imagery involving the lower limbs by the EEG-based brain network. Comput Intell Neurosci. 2021;2021:5565824. 34257636 10.1155/2021/5565824 PMC8245246

[59] Sahiner B, Chan HP, Petrick N, Wagner RF, Hadjiiski L. Feature selection and classifier performance in computer-aided diagnosis: The effect of finite sample size. Med Phys. 2000;27(7):1509–1522. 10947254 10.1118/1.599017 PMC5713476

